# Assessment of hearing performance of dental technicians due to the professional noise exposure

**DOI:** 10.1186/s12903-023-03392-2

**Published:** 2023-09-22

**Authors:** Sunil Kumar Vaddamanu, Fahad Hussain Alhamoudi, Rayan Ibrahim H. Binduhayyim, AlBandary Hassan AlJameel, Maram Ali M. Alwadi, Marco Di Blasio, Marco Cicciù, Giuseppe Minervini

**Affiliations:** 1 Department of Dental Technology, College of Applied Medical Sciences, King Khalid University, 61421 Abha, Saudi Arabia; 2 Department of Periodontics and Community Dentistry, College of Dentistry, King Saud University, 11451 Riyadh, Saudi Arabia; 3 Dental Health Department, College of Applied Medical Sciences, King Saud University, 11451 Riyadh, Saudi Arabia; 4 Department of Medicine and Surgery, University Center of Dentistry,, University of Parma, 43126 Parma, Italy; 5 Department of Biomedical and Surgical and Biomedical Sciences, Catania University, 95123 Catania, Italy; 6 Multidisciplinary Department of Medical-Surgical and Dental Specialties, University of Campania, Luigi Vanvitelli, 80138 Naples, Italy; 7 Saveetha Dental College & Hospitals Saveetha Institute of Medical & Technical Sciences, Saveetha University, Chennai, India

**Keywords:** Noise, Hearing capacity, Dental technicians, Dental laboratory

## Abstract

**Background:**

Some of the noise-intensive processes in dental laboratories include the finishing of crowns, bridges, and removable partial dentures; blowing out workpieces with steam and compressed air; and deflating casting rings. High sound pressure levels are also present in dental vibrators, polishing equipment, and sandblasters. The aim of this study was to Evaluation of the effect of noise production in dental technology laboratory on dental technician hearing capacity.

**Methods:**

For this cross-sectional study, a total of 120 dental technicians were chosen. Otoscopic evaluation and the Weber test were used to establish if they had sensorineural or transmission hearing loss at 500 Hz, 1000 Hz, 2000 Hz, and 4000 Hz, respectively. Then an OAER (objective auditory evoked response) and PTA (clinical aurimeter) test were administered (Neurosoft, Russia). The whole procedure was carried out by an audiologist and an ENT specialist.

**Results:**

The PTA results showed that the patient had mild hearing impairment overall, with the loss being more severe in the left ear than in the right. The OAE test results revealed that in-ear of the left side, 84.5% of subjects passed and 15.5% of subjects struggled and were referred to an ear specialist, whereas in the right ear, 82.7% of subjects passed and 17.3% struggled and were referred to an ear specialist. According to this study, in a right-handed study participant, the ear on the left side is more vulnerable than the right side. Differences in the mean hearing threshold at 4000 and 6000 Hz in the left ear were statistically significant in the groups of workers with eleven to fifteen years of practical experience and twenty-one to twenty-five years of practical experience, respectively (Minervini, et al. J Clin Med 12:2652, 2023).

**Conclusions:**

A statistically meaningful threshold shift from 4000 to 6000 Hz is observed as the working experience grows, and this is suggestive of sensorineural hearing impairment brought on by the noisy dental environment.

## Introduction

The act of perceiving sound is an intensely personal encounter, fundamentally intertwined with the nuances of individual auditory processing. Sound waves, as they traverse the atmospheric medium in the form of radiant energy, culminate in a range of auditory experiences that are as diverse as the listeners themselves [1].

The complex spectrum of auditory perception includes both noise and sound, each with distinct connotations and physiological effects. Noise, often denoted as an unwelcome medley of discordant tones, presents a stark contrast to the perception of sound, which may be regarded as either soothing or disquieting depending on the listener's interpretation. These perceptions oscillate on a subjective pendulum, varying from one individual to another, and underscoring the highly personalized nature of auditory perception [1, 2].

In this intricate labyrinth of sound and noise, what resonates as melodious harmony for one might register as disturbing cacophony for another. Long-standing exposure to what is perceived as noise can instigate not only irritation but potentially trigger a tangible discomfort, even pain [2]. Over time, such enduring auditory assault could precipitate detrimental consequences, with hearing loss being a potential outcome [3].

The profundity of this personal experience underscores the imperative for an increased understanding of the interplay between auditory perception and its long-term effects on our hearing health. It necessitates a thoughtful conversation around the nature of our soundscapes and the impact of prolonged noise exposure, with a view to safeguarding our auditory wellness in an increasingly cacophonous world [3–7]. Statistics Canada has released figures indicating that an estimated 19% of adults, equivalent to around 4.6 million individuals, are dealing with a degree of hearing impairment. This impairment is classified as at least a mild loss of auditory function, specifically within the frequency range that encompasses speech [8]. These statistics highlight the widespread nature of auditory health issues amongst the adult population, pointing out that almost one in five adults experiences challenges in hearing everyday conversation [8]. This illustrates the critical importance of focusing on hearing health and implementing strategies for its preservation and enhancement [9, 10].

Sensorineural hearing loss is characterized by impairment or damage to the structures within the inner ear, resulting in compromised auditory function. It typically involves harm to the hair cells in the cochlea that are instrumental in transmitting sound signals to the brain [8]. The most prevalent form of sensorineural hearing loss is age-related hearing loss, also known as presbycusis. This type of hearing loss naturally occurs as people age and their auditory system undergoes progressive degenerative changes [8]. Especially especially during fixed prosthesis [11–15].

Following presbycusis, noise-induced hearing loss, or NIHL, is the next most common form of sensorineural hearing impairment. NIHL arises from damage to the delicate components of the inner ear due to excessive exposure to loud noises [16]. These noises can be continuous or intermittent but, in either case, their high decibel level is detrimental to the sensitive structures of the inner ear, causing a loss of hearing over time. This highlights the importance of sound regulation and the use of protective measures, especially in loud environments, to preserve our auditory health [8, 17, 18].

Studies in the field of dentistry have revealed that a significant number of dental technicians are at risk of hearing loss [19]. Researchers have also found that the left ear sustains more damage in right-handed dental technicians than in the right ear [20–23].

Some noise-intensive procedures in dental laboratories include finishing crowns, bridges, and removable partial dentures; clearing workpieces with steam and compressed air; and deflasking casting rings [24]. Dental vibrators, polishing equipment, and sandblasters also generate high sound pressure levels. Specifically, the noise level for processing removable partial dentures is around 86 dB, steam jets can exceed 90 dB, and compressed air blasting can reach up to 105 dB. It is important to note that these measurements are based on an eight-hour exposure time or equipment runtime [24–26].

However, it is highly unlikely that a dental assistant would solely grind metal or keep the trimmer running continuously throughout the day. There are typically breaks in the noise, allowing the ears some respite. According to Directive 2058 of the Association of German Engineers (VDI), a daily noise level below 85 dB is generally considered "non-hazardous" even with long-term exposure [27]. The VDI indicates that it takes more than 15 years of exposure at 85 dB(A) and 10 years at 87 dB(A) during eight-hour workdays for hearing damage to occur [28].

Pure-tone audiometry (PTA) is the first test to quantitatively assess hearing loss, allowing for the evaluation of the type and degree of impairment in individuals aged and older. Otoacoustic emission (OAE) testing can also be employed to detect early indications of inner ear anomalies. These hearing assessments can contribute to primary prevention planning and the prevention of hearing loss [18]. At this stage, speech processing is not significantly affected; therefore, without proper testing, the individual might not recognize their condition. If left untreated, hearing loss may progress to the third stage, at which point the person realizes they are losing the ability to hear lower-pitched sounds essential for interpreting speech and seeks medical assistance. Unfortunately, even with medical intervention, the hearing impairment remains irreversible [29, 30].

Despite references in the literature to dental noise-induced hearing loss, there are limited precautions or regulations in place to prevent these disturbances from causing hearing damage in dental laboratories. Consequently, the study aimed to examine the impact of noise levels in dental laboratories on dental technicians' hearing.

### Problem statement

In dental clinics and laboratories, dental technicians are constantly exposed to noise originating from various dental instruments. The effect of chronic exposure to these noise levels on the hearing ability of dental technicians had not been sufficiently explored. The long-term exposure to noise might be affecting the hearing performance of these professional. Leading to potential hearing impairment or loss.

### Aim of the study

This study aims to investigate whether continuous exposure to noise in the dental workplace hasa quantifiable impact on the hearing performance of dental technicians. It will also explore what measures, if any, are being taken to protect dental technician’s hearing and whether they are effective.

### Null hypothesis

There is no statistically significant difference in the hearing performance of dental technicians who are exposed to professional noise.

### Alternative hypothesis

There is statistically significant difference in the hearing performance of dental technicians who are exposed to professional noise.

## Materials and methods

The study was conducted in accordance with the Declaration of Helsinki, and the protocol was approved by the Research Ethics Committee at King Khalid University, Saudi Arabia [ECM#2023–611, following the protocol HAPO-06-B-001]. The study protocol was developed, and all subjects gave their written informed consent for inclusion before they participated in the study. The process of recruitment involved reaching out to potential participants, either through direct phone calls or face-to-face interactions. During these communications, the possible impacts of instrument noise on auditory health were clearly explained to them. The individuals were then invited to participate in this cross-sectional study that seeks to explore the correlation between such noise exposure and potential hearing loss. This proactive approach ensures that potential participants are fully informed about the study's aims, the nature of their involvement, and the potential benefits of the research.

A cohort comprising 120 dental technicians was enrolled to evaluate the prevalence of sensorineural or conductive hearing loss at frequencies of 500 Hz, 1000 Hz, 2000 Hz, and 4000 Hz (Table 1). This sample size of the study was supported by the medium effect size, an alpha of 0.05, a power of 0.80 and population variability parameters.

**Table 1:** Distribution of the sample

**Age**	
20–25	14
26–30	24
31–35	32
36–40	22
41–45	12
46–50	12
51–55	4
**Working experience**	
0–5	16
6–10	52
11–15	22
16–20	12
21–25	12
26–30	6
**Working hours**	
0–5	4
6–10	102
11–15	14

### Inclusion criteria

Participants eligible for inclusion in this study must be dental technicians fulfilling the subsequent criteria:

- Professional experience: A minimum of five years of professional practice in the field of dental technology.
- Age consideration: participants must fall within the age range of 23 to 60 years. This range has been selected to ensure that the sample represents and active working population within the dental technology field.
- Specialization affiliation: eligible technicians must be affiliated with one or more of the following departments with in a dental school or similar professional setting: orthodontics, pedodontics, prosthodontics or periodontics.
- Full time workers: All the participants were full time workers, which defined by those working a standard full time schedule within their respective departments. This because Full-time dental technicians typically work a consistent number of hours each week, allowing for a standardized measurement of noise exposure. This uniformity aids in establishing a more controlled comparison across participants and also as full-time workers are likely to spend more hours in the noise-intensive environment, the cumulative effect on hearing can be more pronounced, making them a more relevant population for the study's focus on hearing loss.

### Exclusion criteria

Participants were deemed ineligible for the study based on the following conditions:

- Use of personal audio devices at High Sound Level: Individuals who consistently utilized an iPod or similar personal audio devices at a sound level of 70% or higher for more than 4 h per day were excluded. This specific exclusion criterion was set to control for potential confounding factors related to non-occupational noise exposure. Such prolonged exposure to high sound levels from personal audio devices could independently affect hearing, making it difficult to attribute any observed effects solely to the professional noise exposure within the dental laboratory setting.
- Recent Experience of Hearing Loss: Participants who had recently experienced hearing loss were also excluded to ensure that the study was measuring the potential effects of chronic occupational noise exposure rather than acute or recent auditory issues. This helped maintain the focus on the potential long-term effects of professional noise exposure in dental technicians.
- Cold or Congenital Ear Diseases: Exclusion of those with a recent cold or congenital ear disease was necessary to avoid the inclusion of individuals with underlying conditions that might independently affect hearing function. These medical conditions could introduce variability into the study results, clouding the interpretation of the specific effects of noise exposure in the dental laboratory environment.
- Tinnitus: Individuals with existing tinnitus were also excluded. Tinnitus can be a symptom of underlying hearing damage or other medical conditions. Including participants with tinnitus might skew the results, as it may not be clear whether the tinnitus or associated hearing loss was caused by occupational noise exposure or other factors.
- Part time worker

To accomplish this objective, participants underwent comprehensive otoscopic examinations and Weber tests. Subsequently, data collection was further supplemented using a clinical audiometer (Edan Ultra sound DUS 60, Shenzhen, China) and an otoacoustic emissions (OAE) test (Ascreen, Neurosoft OAE, Ivamova, Russia).

A team of experienced audiologists and otolaryngologists collaborated in executing the entire diagnostic procedure. The acquired data were subjected to rigorous statistical analysis utilizing Tukey's honest significant difference (HSD) test and two-way analysis of covariance (ANCOVA) for computational and comparative purposes, respectively. The analysis of the data was performed with the assistance of SPSS software, version 23.0 (IBM, USA). To identify results that were statistically meaningful, we set a P-value threshold of less than 0.05. Any results meeting this criterion were considered to demonstrate statistical significance.

## Results

The PTA results indicated that the patient had an overall mild hearing impairment, with the left ear experiencing a more significant loss than the right ear. The OAE test results demonstrated that in the left ear, 84.5% of subjects passed and 15.5% struggled and were referred to an ear specialist. In the right ear, 82.7% of subjects passed and 17.3% struggled and were referred to an ear specialist (Table 2).

**Table 2:** Pure-tone audiometry findings associated with working experience in left ear

Working Experience	PTA (mean ± SD)
0–5 years	16.05 ± 5.96
6–10 years	17.36 ± 5.35
11–15 years	21.61 ± 5.02
16–20 years	15.27 ± 4.79
21–25 years	20.27 ± 4.15
26–30 years	19.44 ± 7.52
F value	0.818
SIG	0.732

**Fig. 1:**
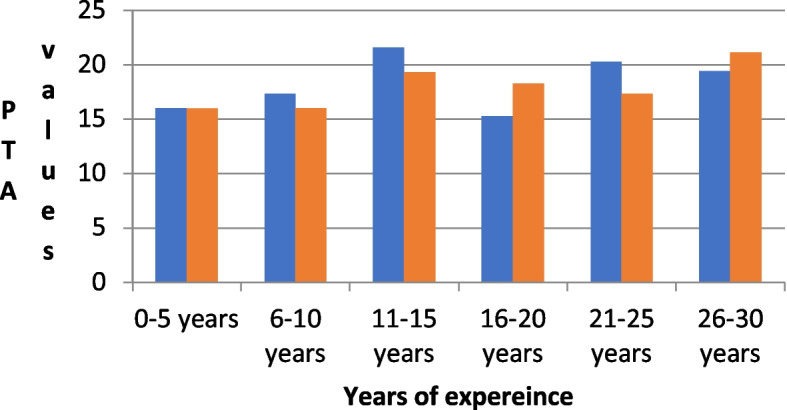
PTA values for the left and right ear with years of experience

For dental technicians with 0–5 years of work experience, the average PTA values were 16.05 ± 5.96 for the left ear and 15.95 ± 7.17 for the right ear. In technicians with 6–10 years of experience, the left ear had an average PTA value of 17.36 ± 5.35, while the right ear had a value of 16.01 ± 5.01. For technicians with 11–15 years of experience, the mean PTA values were 21.61 ± 5.02 for the left ear and 19.31 ± 7.26 for the right ear (Fig. 1 and Table 3).

**Table 3:** Pure-tone audiometry findings associated with working experience in right ear

Working Experience	PTA (mean ± SD)
0–5 years	15.95 ± 7.17
6–10 years	16.01 ± 5.01
11–15 years	19.31 ± 7.26
16–20 years	18.31 ± 3.23
21–25 years	17.36 ± 3.84
26–30 years	21.11 ± 6.55
F value	0.919
SIG	0.742

In dental technicians with 16–20 years of experience, the left ear had an average PTA value of 15.27 ± 4.79, while the right ear had a value of 18.31 ± 3.23. For those with 21–25 years of experience, the left ear had an average PTA value of 20.27 ± 4.15, while the right ear had a value of 17.36 ± 3.84. Lastly, in technicians with 26–30 years of experience, the left ear had an average PTA value of 19.44 ± 7.52, while the right ear had a value of 21.11 ± 6.55.

This analysis suggests that the left ear is less efficient than the right ear in right-handed individuals. Workers with 11–15 years and 21–25 years of experience both exhibited a highly significant average hearing threshold shift at 4,000 and 6,000 Hz in the left ear. Furthermore, a statistically significant difference was observed between the left ear's DP adequacy at 6,000 Hz and 8,000 Hz (mean difference) for individuals aged 51–55 (Table 4).

**Table 4:** Multiple comparisons of age groups using Tukey honestly significant difference test

Frequency	Group (I)	Group (J)	Sig	95% Confidence Interval	
6000 Hz	51–55	20–25	0.00	16.25	62.32
		26–30	0.00	12.23	56.11
		31–35	0.00	14.08	14.08
		36–40	0.01	9.74	53.90
		41–45	0.01	11.54	58.46
		46–50	0.02	9.04	55.96
8000 Hz	51–55	20–25	0.00	16.61	56.96
		26–30	0.00	16.79	55.48
		31–35	0.00	17.07	54.81
		36–40	0.00	15.88	54.57
		41–45	0.00	16.12	57.21
		46–50	0.00	14.46	55.54

## Discussion

As a fundamental facet of human existence, the phenomenon of sound exerts a profound influence on our daily lives, often acting as a catalyst for an extensive array of emotional responses. It is a pervasive element of our experiential reality, a sensorial stimulant capable of kindling profound affective experiences across a wide emotional spectrum. It's essential to note that this spectrum isn't exclusively positive; auditory stimuli can also evoke negative emotional responses [31].

In the wider auditory milieu, there exists a counterpoint to the harmonious cadence of sound: the discordant cacophony of noise. Generally depicted as an amalgamation of inharmonious, unsettling auditory elements, noise has the potential to incite irritation or distress. Often characterized as unwanted sound, noise infiltrates our auditory landscape, occasionally clashing with our quest for aural serenity [32].

Yet, the demarcation lines separating sound and noise are nebulous and fluid, intrinsically subjective in nature. An array of sonic frequencies might engender disparate reactions among different individuals. In an intriguing twist of subjective perception, a frequency sequence that serves as a melodic lullaby to one set of ears may be an irksome disturbance to another. This contrast underlines the deeply personal, subjective nature of auditory perception [33].

Thus, our auditory environment is a complex and dynamic tapestry woven from myriad auditory threads, its intricate patterns often shaped by our individual perceptual and emotional responses. This intricate interplay between sound and noise, between harmony and discord, serves as a constant reminder of the profound influence that our auditory surroundings exert on our personal and collective experiences [34].

Occupational Noise-Induced Hearing Loss (ONIHL) constitutes a significant health concern, notably affecting an individual's auditory capabilities. It is distinguished by a gradual, unyielding encroachment of bilateral sensorineural hearing impairment, evolving incrementally yet relentlessly over a protracted duration, often spanning multiple years [35].

ONIHL, a specific type of hearing loss, doesn't develop in a spontaneous or capricious manner; rather, its onset is an intricate process that accumulates over prolonged timeframes. The critical causative factor is typically the individual's enduring or intermittent exposure to excessive decibel levels within their vocational milieu [35, 36].

The term "noise" in this context is perhaps a misnomer, for the auditory stimuli in question far surpass the conventional parameters of loudness. Instead, these pervasive sounds eclipse the realms of normalcy or safety, entering the territory of potential auditory trauma, gradually eroding the individual's hearing acuity [35, 37].

This particular occupational peril accentuates the cardinal role of adequate auditory protective measures and rigorous noise regulation within working environments. Such preventative strategies are particularly pertinent in settings inherently characterized by high-decibel operations. The insidious nature of ONIHL, coupled with the potential for irreversible auditory damage, amplifies the urgency for implementing comprehensive noise mitigation and hearing preservation measures in workplaces prone to elevated sound levels [3].

Therefore, the topic of ONIHL necessitates broader discourse and practical interventions, given its widespread implications for the workforce's auditory health and overall quality of life. The establishment of preventive measures, increased awareness, and timely interventions can significantly attenuate the adverse impacts of this pervasive occupational hazard [38].

Hearing impairment that results from the natural aging process or is determined by genetic predispositions unfortunately remains beyond the realm of prevention. However, an entirely different scenario presents itself when we consider noise-induced hearing loss. This specific type of hearing damage, which arises due to consistent exposure to loud environments, can indeed be effectively prevented. One can guard against such auditory damage by utilizing appropriate protective gear in environments characterized by high noise levels. This includes the use of specific auditory protection devices such as ear plugs and ear muffs. These tools serve as an effective barrier, significantly reducing the level of noise that reaches the eardrum, thereby mitigating the risk of noise-induced hearing damage. By integrating such protective measures, we can effectively safeguard our auditory health in noise-intensive situations [39].

Long-term exposure to such noise, particularly in occupational settings, can cause significant discomfort and may ultimately lead to hearing loss. Numerous studies in the field of dentistry have revealed that a substantial proportion of dental technicians are at risk of developing hearing loss, often beginning around the age of 35 [20, 21].

This susceptibility to hearing loss can be attributed to various factors, including the nature of the dental work environment, which often involves high noise levels from equipment and machinery. Dental technicians are exposed to these noise levels on a regular basis, and without proper precautions, the constant exposure can contribute to the gradual deterioration of their hearing capabilities [38].

Despite the literature linking noise in dental laboratories to hearing loss, there are limited protective measures or regulations in place to mitigate its occurrence. Consequently, this study aimed to evaluate the impact of noise on the hearing of dental laboratory employees. The Pure Tone Audiometry (PTA) results revealed mild hearing impairment overall, with greater severity in the left ear. The Otoacoustic Emissions (OAE) test results demonstrated that 84.5% of subjects in the left ear passed, while 15.5% struggled and were referred to an ear specialist. This outcome aligns with earlier investigations by Zubick et al. and Alabdulwahhab et al. [25, 40], which found that right-handed dental professionals experienced a higher degree of hearing loss in their left ear.

To determine personal noise exposure in the laboratory, one must calculate the daily exposure level. The Institute for Prevention and Occupational Medicine of the German Social Accident Insurance [41] provides a noise exposure calculator for this purpose. The calculator allows the input of work tasks, noise levels, and durations, either online or offline. The result displays the total sum of individual noise events over eight hours, calculated using a logarithmic function rather than simple addition. A study by the Institute for Occupational Safety and Health (BIA) in 2003 was the first to investigate the risk of noise-induced hearing damage among dentists and dental technicians. Noise measurements were conducted at three practices in Cologne and seven dental laboratories. The findings revealed that the location-based daily sound pressure for dental technicians was approximately 68 dB(A), and the personal level was around 76 dB(A). Although there were occasional instances exceeding 80 dB(A), no health-damaging values were measured [42]. Dr. Tilman Brusis [43] and his research team concluded that significant hearing damage, impairing the ability to hear and understand speech, was unlikely to occur due to noise exposure in dental technicians and dentists.

The analysis showed that the left ear was less effective than the right ear in right-handed individuals. Workers with 11–15 years and 15–25 years of experience exhibited a significantly different mean hearing threshold shift at 4000 Hz and 6000 Hz in the left ear. Additionally, a statistically significant difference was observed in the left ear's Distortion Product (DP) amplitude at 6000 Hz and 8000 Hz for those aged 51–55.

In 2016, Dr. Brusis [19] reiterated that noise-induced hearing loss in the context of occupational disability could be ruled out in dental practices and laboratories under normal circumstances. Dental equipment manufacturers have been striving to reduce noise for several years, continually working towards achieving lower sound pressure levels.

Despite these efforts, noise in the laboratory can be bothersome and cause long-term stress. In an online survey conducted by the Association of Medical Professions in April 2019, 71.5% of the 1,170 dental technicians assessed their work stress on a scale of zero (low) to ten (very high) as ranging between seven and ten [44]. Nearly three-quarters of respondents experienced psychological stress during their work in the laboratory. The primary stressor was time pressure (average assessment of 7.78), followed by workload (7.31), and in third place, physical stress.

This indicates that nearly three-quarters of the respondents experienced significant psychological stress during their work in the laboratory. For self-employed master dental technicians, this figure reached 73 percent, while for apprentices, it was already 56.5 percent. The primary reason cited for this high stress was time pressure (average rating 7.78), followed by workload (7.31), and in third place, physical stress resulting from noise, dust, prolonged sitting, and work involving microscopes, among other factors (7.03). This demonstrates that noise in the laboratory is not only a nuisance but also a significant source of stress for dental technicians [45].

Although the noise exposure in dental laboratories is typically not high enough to pose a risk of hearing loss, it is still advisable to minimize noise exposure. Noise-induced stress can trigger the release of hormones such as adrenaline, noradrenaline, and cortisol, potentially leading to increased blood pressure and reduced concentration and work quality. Prolonged exposure may also cause headaches, muscle tension, digestive issues, and sleep disturbances [21]. As a result, those considering purchasing new equipment for their laboratories should examine noise emissions and frequencies. Individual hearing protection can also help alleviate noise-induced stress. By soundproofing the CAD/CAM area and installing milling machines, 3D printers, and other noise-intensive equipment in separate rooms, practitioners can further contribute to noise reduction in their laboratories.

Previous research on the decibel levels of various dental handpieces and equipment reached similar conclusions, with laboratory instruments producing the highest decibel levels (up to 85.3 dB). OSHA regulations stipulate that an 8-h daily exposure to noise levels of 85 dB is acceptable [21]. Dentists typically do not operate high-speed handpieces continuously for more than eight hours per day, with most individuals using high-speed handpieces sporadically for 15 to 30 s. This finding might suggest a low likelihood of experiencing noise-induced hearing loss (NIHL) due to dental drills alone. However, another study discovered that high-speed handpieces generate sound at wavelengths that could ultimately lead to hearing damage. Although these figures may be below the 85 dB OSHA maximum permissible value, caution should be exercised when interpreting them, as prolonged exposure to these noise levels can be harmful [46]. The noise level measured in the dental laboratory exceeded the 85 dB maximum permissible value, raising concerns given that dental technicians spend 6 to 8 h daily in the dental laboratory, placing them at high risk.

The dental stone cutter (92.0 dB) was identified as the loudest piece of equipment in the dental lab, while the denture-polishing unit was the quietest (41.0 dB) [24]. Continuous exposure to noise levels above 85 dB results in acute hearing impairment. Additionally, long-term hearing loss can occur due to repeated exposure to extremely loud noise levels caused by blasts or explosions near the ear [47]. Although the highest noise exposure in dental offices falls below the threshold causing hearing loss in humans, it remains dangerously close to that limit (85.0 dB) [48]. Kryter asserts that irreversible hearing loss can be expected after exposure to noises with frequency components above 80 dB for eight hours a day, five days a week [28]. High-speed dental air turbines have been identified as a leading cause of permanent hearing loss, according to research by Altinöz et al. [49]. Some experienced dentists have trouble hearing at high frequencies, as noted in a long-term study by Taylor et al. [29].

It is widely established in scientific literature that exposure to noise levels exceeding 85 decibels for extended periods, especially without the use of any form of ear protection, can lead to hearing loss. Such instances of noise-induced hearing loss are well-documented, thereby accentuating the risk posed by high-decibel environments [50–52]. Consequently, the noise produced in settings such as dental clinics should not be taken lightly or underestimated. Despite the commonplace nature of such noises in the dental profession, they carry potential risks for long-term auditory health [10, 40]. This underlines the necessity of implementing suitable preventive measures and protective strategies in such occupational settings to safeguard against potential hearing damage.

Hearing is indeed an intimate process, as sound waves travel through the air. Pure-tone audiometry (PTA) is the first statistically quantifiable test for hearing loss and can assess the type and degree of impairment in individuals aged four and older. Otoacoustic emissions (OAE) testing can also detect early signs of inner ear abnormalities [46]. These hearing tests can be beneficial for both primary prevention and the prevention of hearing loss. If proper testing is not initiated, a person may not recognize their condition since speech processing is not significantly affected at first [21]. Hearing loss may progress to the third stage before a person seeks medical help, realizing they are losing the ability to hear lower-pitched sounds essential for understanding speech [21]. Unfortunately, there is currently no way to reverse the hearing loss, even with medical treatment.

In the context of this study, standardization of the exposed noisy environment for each participant was achieved through rigorous controls and procedures. We ensured that all participants were exposed to similar noise levels by monitoring and calibrating the equipment consistently across different laboratory settings. Additionally, all dental technicians were instructed to follow the same protocols, and we used precise instruments to measure the noise levels to which each participant was exposed.

However, it is important to acknowledge that complete standardization of noise exposure in a real-world laboratory setting can be challenging. Variability in individual work practices, slight differences in equipment across laboratories, and the influence of other environmental noise factors could introduce some degree of variation. While every effort was made to minimize these potential sources of discrepancy, they might still represent a limitation of the study. Future research could focus on further refining the standardization process or employing additional controls to mitigate this limitation.

## Conclusions

In conclusion, this study underscores the potential risk of hearing loss in dental technicians due to prolonged exposure to noise in dental laboratories. While the noise levels generally do not exceed the hearing loss threshold, consistent exposure can lead to a gradual decline in hearing, predominantly in the left ear of right-handed individuals. However, it's worth noting that the standardization of the exposed noisy environment for each participant may present a limitation, as complete control over individual work practices and environmental noise factors may vary. Despite this, the research emphasizes the urgent need for increased awareness, protective measures, and regulations, including improved equipment design, individual hearing protection, and soundproof work areas to mitigate the risk of noise-induced stress and long-term hearing impairment.

## Data Availability

Dr. *Sunil Kumar Vaddamanu* will have access to the data that were the basis for this article, and can be reached out for data in case is needed for review.

## Abbreviations

OAER: Objective auditory evoked response
PTA: Pure-tone audiometry
NIHL: Noise-Induced Hearing Loss
dB: Decibel
Hz: Hertz
OAE: Otoacoustic emissions
ONIHL: Occupational Noise-Induced Hearing Loss
CAD/CAM: Computer-Aided Design" and "Computer-Aided Manufacturing
3D: 3 Dimensional
OSHA: Occupational Safety and Health Administration
